# The Crotone Megalandslide, southern Italy: Architecture, timing and tectonic control

**DOI:** 10.1038/s41598-018-26266-y

**Published:** 2018-05-17

**Authors:** Massimo Zecchin, Flavio Accaino, Silvia Ceramicola, Dario Civile, Salvatore Critelli, Cristina Da Lio, Giacomo Mangano, Giacomo Prosser, Pietro Teatini, Luigi Tosi

**Affiliations:** 1 (OGS) Istituto Nazionale di Oceanografia e di Geofisica Sperimentale, Borgo Grotta Gigante, 42/c, 34010 Sgonico, Trieste Italy; 2 Dipartimento di Biologia, Ecologia e Scienze della Terra, Università della Calabria, 87036 Arcavacata di Rende, CS Italy; 3 Institute of Marine Sciences, National Research Council, Arsenale - Tesa 104, Castello 2737/F, 30122 Venezia, Italy; 4 Dipartimento di Scienze, Università della Basilicata, Potenza, Italy; 5 Department of Civil, Environmental and Architectural Engineering, University of Padua, via Marzolo 9, 35121 Padova, PD Italy

## Abstract

Large-scale submarine gravitational land movements involving even more than 1,000 m thick sedimentary successions are known as megalandslides. We prove the existence of large-scale gravitational phenomena off the Crotone Basin, a forearc basin located on the Ionian side of Calabria (southern Italy), by seismic, morpho-bathymetric and well data. Our study reveals that the Crotone Megalandslide started moving between Late Zanclean and Early Piacenzian and was triggered by a contractional tectonic event leading to the basin inversion. Seaward gliding of the megalandslide continued until roughly Late Gelasian, and then resumed since Middle Pleistocene with a modest rate. Interestingly, the onshore part of the basin does not show a gravity-driven deformation comparable to that observed in the marine area, and this peculiar evidence allows some speculations on the origin of the megalandslide.

## Introduction

Kilometer- to tens of km-scale submarine gravitational collapses, here referred to as megalandslides, consist of land movements involving up to 1 km (or even more) thick sedimentary successions sliding on a basal surface that may classify as ‘basal overpressured shale detachment’ or ‘salt detachment’^1^. Gravity gliding may be instantaneous, associated with mass wasting and shallow detachment, or slow, in connection with a deep detachment and long-term geological processes such as high sedimentation rates or uplift in adjacent areas^1^. These large-scale phenomena typically produce an updip extensional domain and a downdip contractional domain, which are linked via a basal detachment surface^2,3^. The interest for megalandslides has involved also the hydrocarbon industry, as they may be associated with structural traps consisting of large anticlines^1^.

Several examples were reported worldwide, mostly but not exclusively on passive margins, such as in the Bight Basin, Australia^4^, Niger Delta^5–7^, West African margins^8–10^, Gulf of Mexico^11–13^, Brazil^14^, Antarctic Peninsula^15^, and Brunei^16^. European examples (see Canals, *et al*.^15^ and references therein) include the Storegga, Trænadjupet and Finneidfjord slides (Norwegian margin), BIG’95 Slide (off the Ebro river, Spain), Central Adriatic Deformation Belt (Adriatic margin, Italy), and Afen Slide (Faeroe-Shetland Channel).

**Figure 1:**
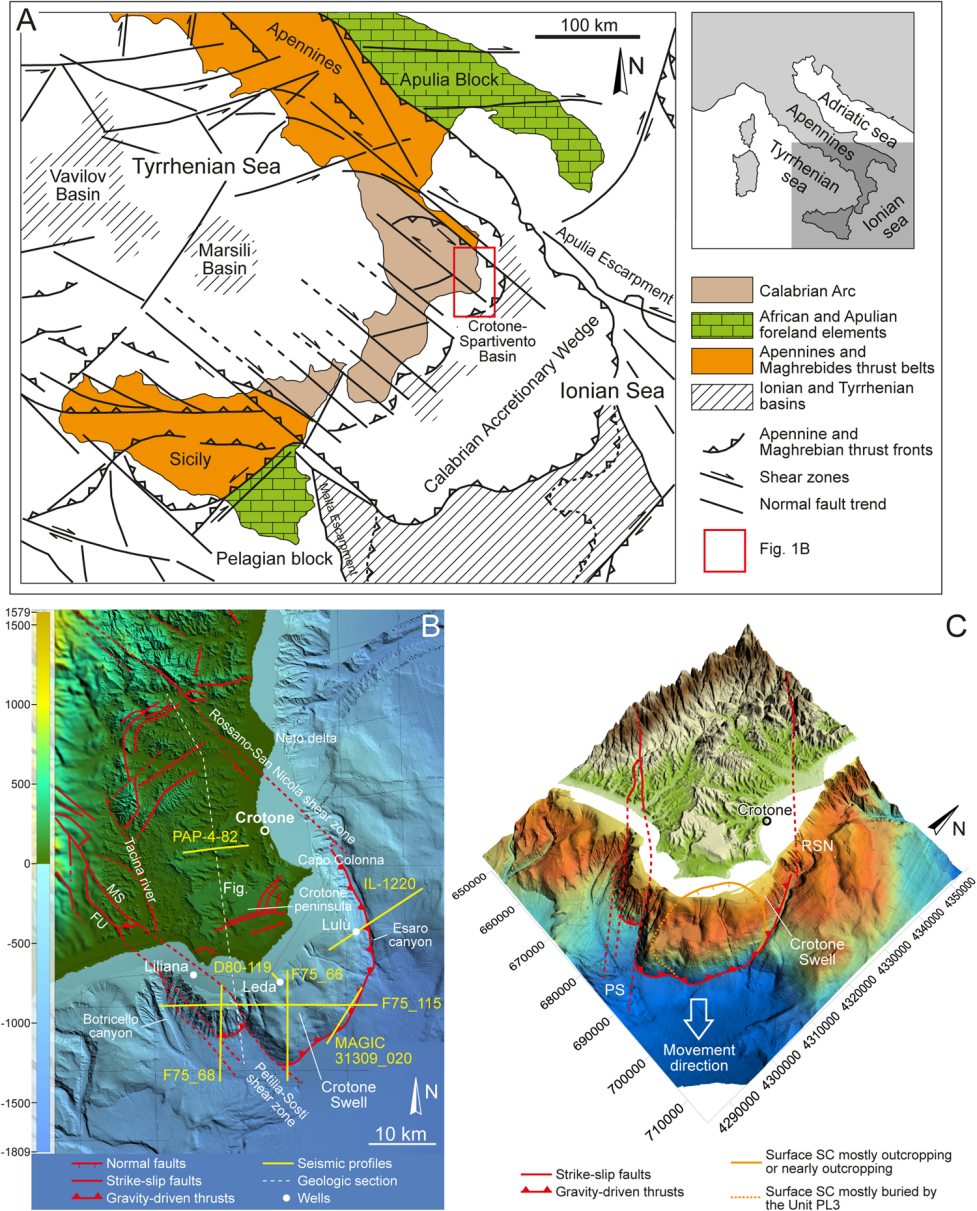
(**A**) Structural map of the Calabrian Arc, which is comprised between the southern Apennines and Sicily (modified from Van Dijk & Okkes^24^). The main structural elements and the Ionian and Tyrrhenian basins are highlighted. (**B**) DTM map showing the onshore and offshore parts of the Crotone Basin (see Fig. 1A for location). The selected seismic profiles and wells are shown. The illustrated faults are reported from various authors^27,30,31,38^. Abbreviations: FU and MS – Fosso Umbro and Marcedusa-Steccato faults. (**C**) Three-dimensional view of the Crotone basin highlighting the Crotone Swell, the extent of the recognized scar (surface SC, see text), and the thrust front showing SE-ward vergence (v. ex. 7×). In (**B**) and (**C**), bathymetric data were acquired by OGS in the frame of the MaGIC (Marine Geohazards along the Italian Coasts) project; public DTM data are available from https://www2.jpl.nasa.gov/srtm/.

The existence of a previously unknown, ca. 1,000 km^2^ megalandslide in the Neogene Crotone Basin, southern Italy (Fig. 1A,B), was recently suggested by Minelli, *et al*.^17^ on the basis of seismic, well and GPS data and literature information. The Crotone Basin is interpreted as a forearc basin located on the Ionian side of the Calabrian Arc (Southern Italy) (Fig. 1A); it is partly exposed in the Crotone area (Fig. 1B,C) and it is also widely documented offshore. The Calabrian Arc is interpreted as a composite terrane that migrated to the SE since the Middle Miocene in response to the subduction of the Ionian oceanic crust^18–22^. Migration was facilitated by the formation of major NW-trending shear zones, which are particularly frequent in the NE sector of the Calabrian arc and represent the NE and the SW boundaries of the Crotone Basin^23–25^ (Fig. 1A,B). The sedimentary infill of the basin consists of Serravallian to Middle Pleistocene marine, coastal and continental deposits, which record main tectonic events and glacio-eustasy^26–31^. Since the Middle Pleistocene, the basin has undergone rapid uplift with an average rate of ca. 1 mm/yr^32^.

The interpretation of Minelli, *et al*.^17^ indicates that most part of the Messinian to Plio-Pleistocene succession of the Crotone Basin (both onshore and offshore) is gliding toward the Ionian Sea above a Messinian halite layer. Evidence of large-scale gravity tectonics affecting Pliocene strata was already reported in previous studies^33^. The megalandslide would be characterized by a downdip contractional domain located offshore and corresponding to the prominent Crotone Swell (Fig. 1B,C), and an onshore updip domain, represented by some seaward-dipping normal faults found in the northern part of the basin^17^ (Fig. 1B).

However, many uncertainties remain about the origin, timing, extent and existence itself of the large-scale gravitational phenomenon involving the Crotone Basin. This generalized uncertainty is highlighted by the different interpretation provided by other authors about the nature of the Crotone Swell^34^, and by the modest deformation and overall good preservation of the Messinian to Plio-Pleistocene succession in the onshore part of the basin, which even contains stratotypes^27,28^. Much more evidence is therefore necessary to address this question. Moreover, the existence of a still active large-scale gravitational collapse involving the onshore part of the basin would have social impacts, as it would raise security concerns about the population of this area, where the city of Crotone (64.000 inhabitants) is located.

The present study is aimed at resolving the existence, areal extent and timing of the Crotone Megalandslide. This has been achieved by integrating on- and offshore datasets acquired across the Crotone Swell and in the onshore part of the basin, including seismic, morpho-bathymetric, sediment cores, outcrops and land movements.

## Results

### The offshore area

South of the Crotone peninsula, the Crotone Swell, a morphological high 16 km long and 30 km wide, is characterized by a prominent lobate morphology with an undulated longitudinal profile (Fig. 1B,C). The high includes a portion of continental shelf from 5 to 10 km wide. The continental slope is subdivided in an upper slope characterized by a flat, NE-SW elongated intraslope basin (at ca. 600 m water depth), and a rather steep (up to 11°) lower slope from 750 m to 1350 m water depth, which is incised by several single and multiple scars up to 5 km wide showing ‘fresh’ morphologies. The Crotone Swell is bounded by two well incised and elongated canyon systems: the Botricello, a quite short (40 km) NW-trending system and the Esaro, a NS-trending asymmetric system developing along the eastern side of the morphological high (Fig. 1B,C).

**Figure 2:**
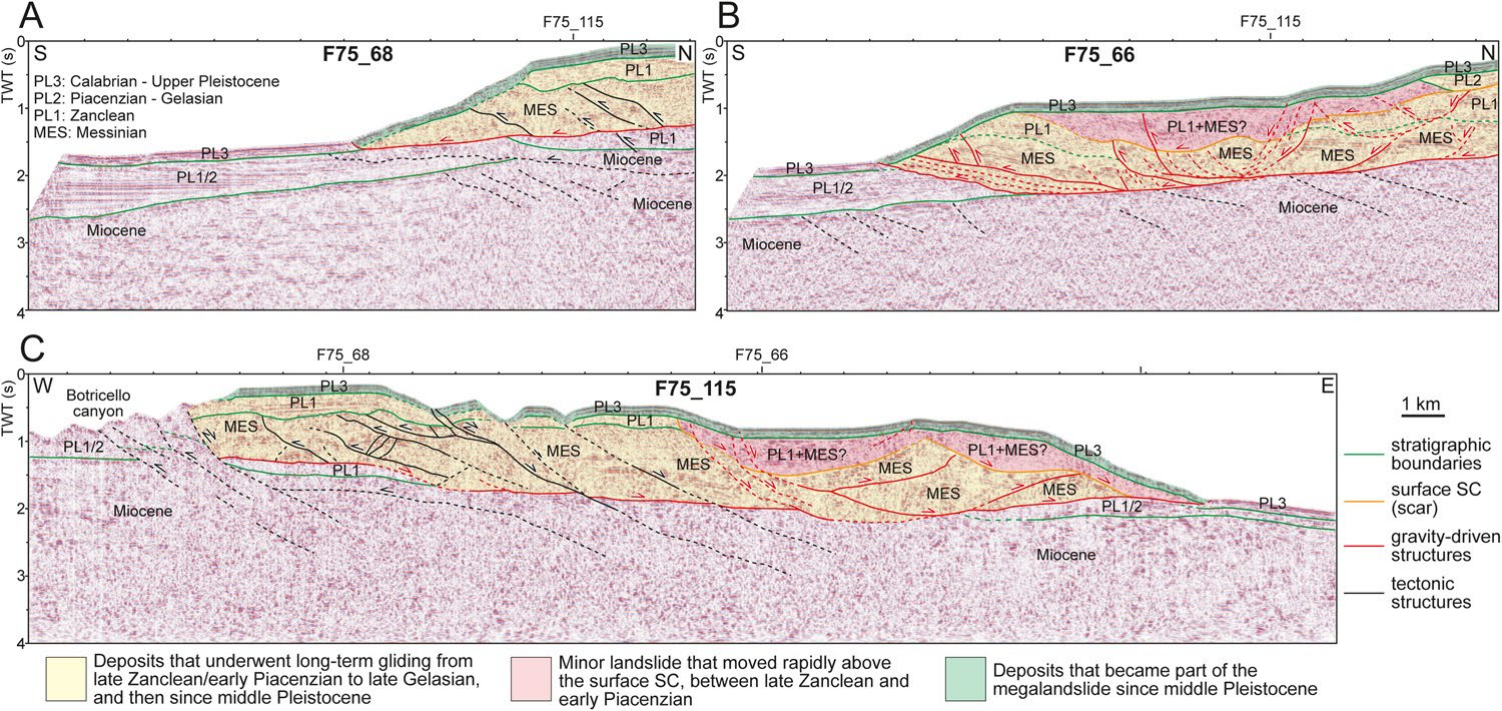
(**A**,**B**,**C**) Interpreted seismic profiles that cross-cut the Crotone Swell (Fig. 1B for location), interpreted to as a megalandslide. SW-verging tectonic structures (in black) are truncated by SE-verging, inferred gravity-driven structures (in red), which are dominated by a basal detachment surface and a thrust front that reaches the seafloor. The gravity-driven phenomenon involves Late Miocene and Plio-Pleistocene deposits, which in seismic profiles correspond to seismic units (Units MES and PL1-3) separated by unconformities. The ages of the seismic units are derived from the available wells (Fig. 5). A minor landslide of inferred mid-Pliocene age is nested in the megalandslide and is bounded at the base by a scar (surface SC). The timing of the gravity-driven process (see areas with different color) is discussed in the text. Seismic data from “Visibility of Petroleum Exploration Data in Italy” (ViDEPI Project) (http://unmig.sviluppoeconomico.gov.it/videpi/). Uninterpreted seismic profiles are available in Supplementary Material Fig. S1.

**Figure 3:**
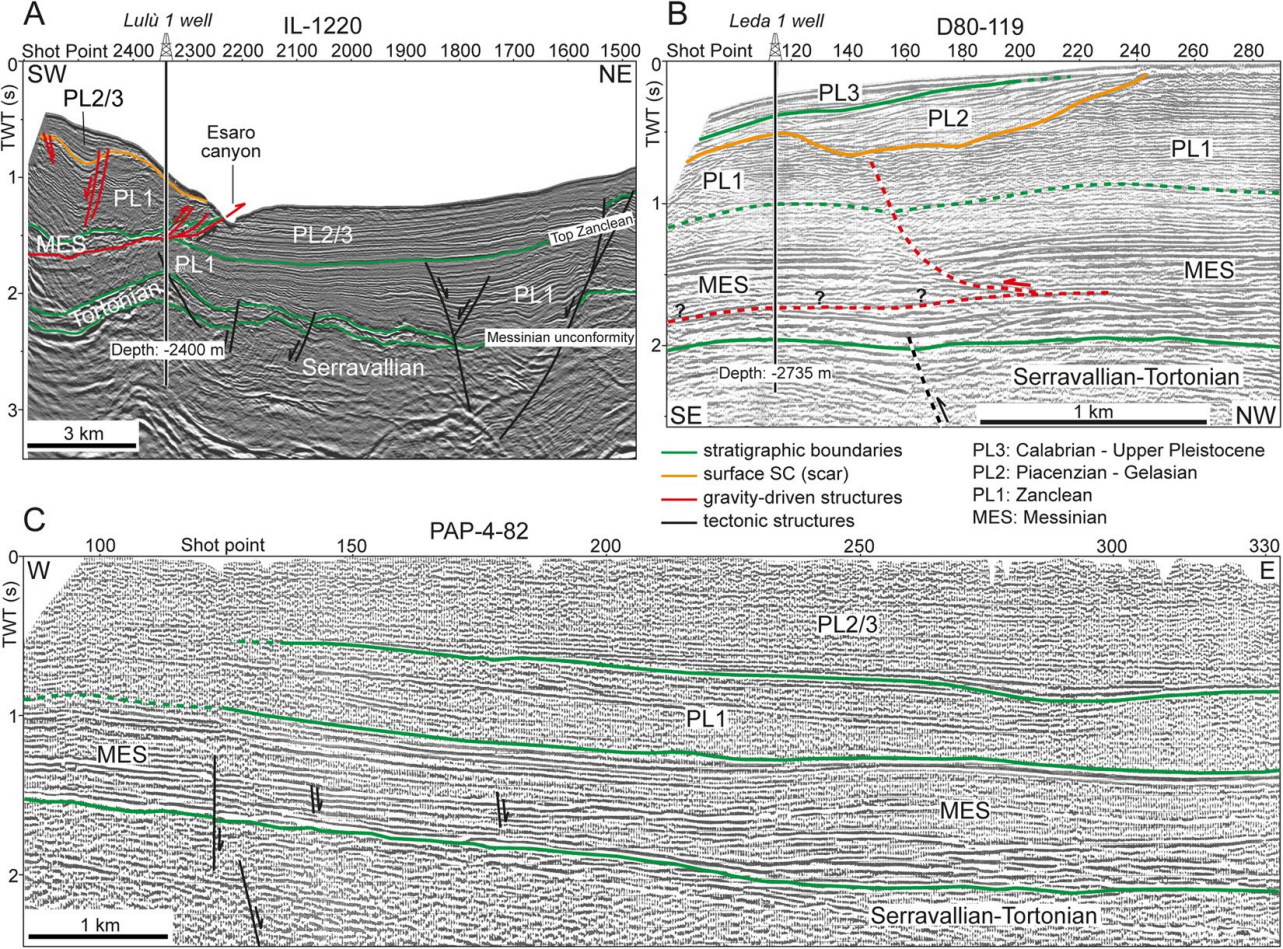
(**A**) The IL-1220 seismic profile, which cross-cut the eastern margin of the Crotone Swell (modified from Zecchin, *et al*.^30^; Fig. 1B for location). Note that the thrust front seems to reach the western wall of the Esaro canyon. The stratigraphy of the Lulù 1 well is shown in Fig. 5. (**B**) The D80-119 seismic profile, which is close to the northern part of the F75_66 seismic profile (modified from Zecchin, *et al*.^30^; Fig. 1B for location). Note the proximal part of the scar (surface SC) that truncates the Zanclean deposits (Unit PL1) and is in turn overlain by undisturbed Piacenzian and Gelasian deposits (Unit PL2). Note also the uncertain detection of the basal detachment surface, which is visible just to the south (see Fig. 2B). The stratigraphy of the Leda 1 well is shown in Fig. 5. (**C**) The PAP-4-82 seismic profile, which is located onshore (modified from Zecchin, *et al*.^27^; Fig. 1B for location). Note the nearly undisturbed sedimentary succession that characterizes the central part of the Crotone Basin. Seismic data from “Visibility of Petroleum Exploration Data in Italy” (ViDEPI Project) (http://unmig.sviluppoeconomico.gov.it/videpi/).

**Figure 4:**
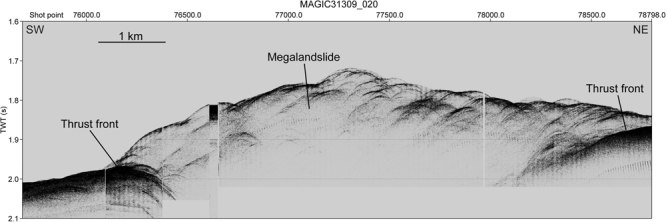
Subbottom profile cross-cutting the front of the megalandslide (Fig. 1B for location). Note that the basal thrust front reaches the seafloor, suggesting modern or recent activity. The profile has been acquired during the MaGIC (Marine Geohazards along the Italian Coasts) geophysical campaign onboard of the R/V OGS Explora in April 2009.

**Figure 5:**
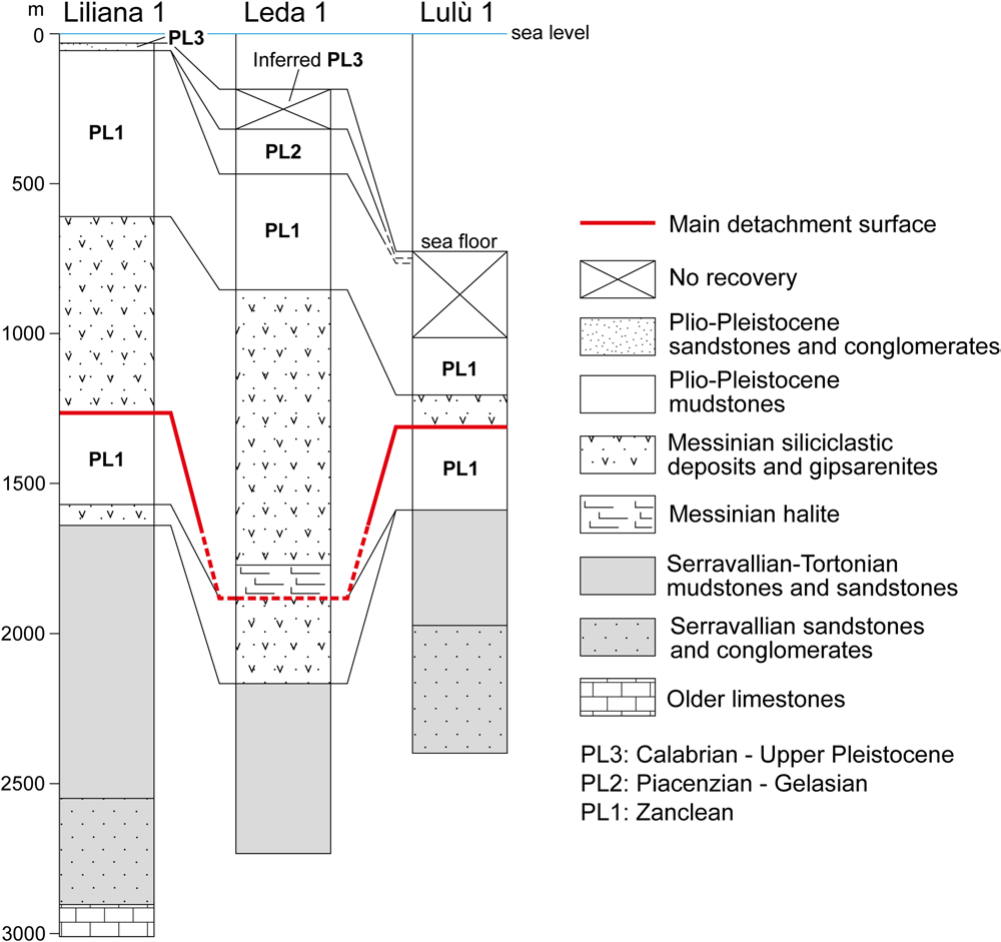
Simplified stratigraphy of the three wells considered in this study (Fig. 1B for location). PL1-3 correspond to the seismic units recognized in the seismic profiles (Figs 2 and 3). Data from “Visibility of Petroleum Exploration Data in Italy” (ViDEPI Project) (http://unmig.sviluppoeconomico.gov.it/videpi/).

The Crotone Swell exhibits a complicated structure, consisting of an offshore (SE-ward) verging thrust, locally passing updip, via a seaward-dipping detachment surface inclined ca. 3° to 4°, into SE-ward-dipping extensional faults (Figs 1B,C, 2 and 3A). Orientation of tectonic structures can be easily ascertained by correlating them in differently oriented seismic lines (Fig. 2). The main contractional structure intersects the seafloor at the base of the slope (Figs 1C, 2, 3A and 4), whereas the basal detachment surface truncates older NE-dipping and SW-verging thrusts (Fig. 2). Minor contractional and extensional structures involve the main body, which is up to 1.5 s TWT (ca. 1.6 km) thick and extends for an area of ca. 25 × 15 km (Figs 1B,C and 2). Based on the available wells, the sedimentary succession that composes the Crotone Swell above the basal detachment surface consists of evaporites and clastic sediments of Messinian age, and of Plio-Pleistocene deep-marine deposits (Fig. 5). Near the thrust front, the basal detachment surface places this succession above either Miocene or Plio-Pleistiocene deposits, depending on the trajectory of the thrust surface or on the overall geometry of the footwall strata (Figs 2 and 3A). The amount of shortening produced by thrusting cannot be ascertained with precision, but it is in the order of several kilometers (at least 10 km based on the F75_68 seismic profile; Figs 1B and 2A), whereas a minor amount of extension, in the order of some hundreds of meters, is associated with the recognized normal faults (Fig. 2B).

In W-E trending transects (Fig. 2C), the SE-ward directed thrust sheet seems to interfere with NW-trending, NE-dipping faults that are inferred to be part of the major Petilia-Sosti regional shear zone^24^ (Fig. 1B), which was characterized by alternating sinistral and dextral transpressional and transtensional activity during the Plio-Pleistocene times^31^. In particular, two main branches of the Petilia-Sosti shear zone are recognizable in the F75_115 seismic profile (Fig. 2C); they likely represent the seaward extension of the Marcedusa-Steccato and Fosso Umbro faults identified onshore (Fig. 1B). The late middle Pleistocene activity of these faults has been interpreted as related to their reactivation as dextral transfer faults during formation of local extensional and contractional gravity-driven structures, connected to SE-directed seaward gliding of part of the sedimentary infill of the Crotone Basin^31^. Following this interpretation, it is inferred that reactivation of the NW-trending structures drove the SE-ward movement of the whole Crotone Swell toward the Ionian Sea (Fig. 1B,C). This observation allows discriminating the structures related to the SE-ward movement of the Crotone Swell from older contractional structures, which show a SW-ward vergence and either are sealed by a surface representing the top of the Messinian succession or were active during Pliocene time (Fig. 2). It is also to be noted that just east of the Petilia-Sosti shear zone, the thrust front is shifted of ca. 8 km to the SE with respect to the thrust segment that is comprised between the seaward parts of the Marcedusa-Steccato and Fosso Umbro faults, where the F75_68 seismic profile has been acquired (Fig. 1B,C).

Seismic profiles document that an irregular, composite surface (surface SC), sealed by undeformed Piacenzian to Gelasian deposits (Unit PL2), truncates the Zanclean succession (Unit PL1) landward with respect to the main thrust front and tends to approach the seafloor toward the shoreline and along the eastern slope of the Crotone Swell (Figs 1C, 2B,C and 3A,B). This irregular surface may be followed downdip in the SE part of the Crotone Swell, where it truncates also Messinian deposits and is overlain by a deformed landmass up to 0.7 s TWT (ca. 0.7 km) thick (F75_66 and F75_115 seismic profiles; Fig. 2B,C). The F75_66 seismic profile documents that the reflectors of the undeformed Unit PL2 onlaps or downlaps the landward part of the deformed landmass (Fig. 2B). These observations are consistent with the presence of deposits not younger than Zanclean (Units PL1 and MES) within the deformed landmass (Figs 2B,C and 3A,B). The landmass was in turn dissected by both extensional and contractional structures (Fig. 2B) that possibly propagated from reactivation of pre-existing faults in the deeper part of the Crotone Swell and/or by newly formed structures that crosscut the whole succession.

All the described features are sealed by a sedimentary drape up to 0.15 s TWT (ca. 150 m) thick of inferred Calabrian to late Pleistocene age (Unit PL3; Figs 2 and 3B), which, excepting for the main thrust at the base of the modern slope, at the seismic scale is affected by only minor faults with negligible offset, probably produced by relatively recent reactivation of older faults. The surface bounding the base of the Unit PL3 is associated with a hiatus that increases seaward. This encompasses the whole Pliocene and Early Pleistocene (Gelasian) in the lower part of the modern slope (Fig. 2B). At the base of the modern slope, the Unit PL3 is affected by a thrust that produces a shortening not greater than 500 m (Fig. 2). Along the relatively steep portion of the slope, the Unit PL3 exhibits local irregular reflections, possibly due to gravitational collapses (Figs 2A and 4).

### The onshore area

In the onshore sector of the Crotone Basin, Serravallian to Middle Pleistocene continental to deep-marine deposits lie in an area that extends for ca. 45 × 30 km (Fig. 1B). SSE- and SE-ward dipping normal faults found in the Crotone peninsula and in the northern part of the Crotone Basin (Fig. 1B), show vertical displacements ranging from m- to hm-scale and lengths up to 10 km^27,35^. Part of these faults are thought to have been originated by gravity-driven processes during both Pliocene and Pleistocene times^27,31^. Salt diapirs and related tectonic structures were also recognized in the northern part of the basin^27,36^. However, seismic profiles (Fig. 3C) and field observations in the onshore portion of the basin do not document the presence of a mega-detachment involving the Messinian to Pleistocene succession such as to justify the km- to tens of km-scale displacement visible offshore.

**Figure 6:**
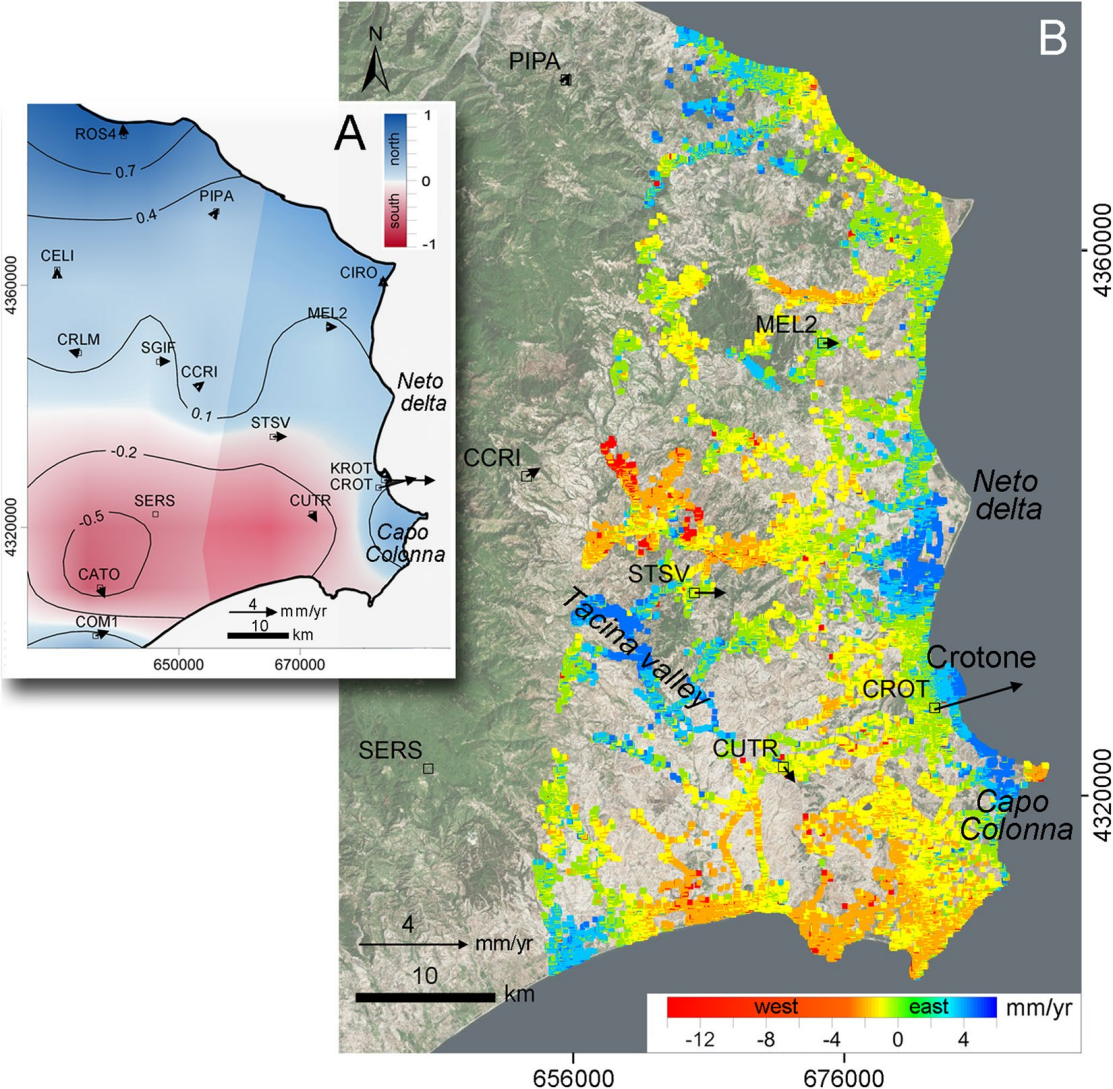
Maps of the ground movements. (**A**) GPS velocities superposed to the interpolated map of south-north displacements referred to SERS. (**B**) Average west-east ground movements obtained by SAR-based interferometry over the 2011–2014 period. Black squares show the positions of the GPS stations, with the arrows providing the direction and magnitude of the horizontal displacement rate. Satellites base map used is from Esri, DigitalGlobe, GeoEye, Earthstar Geographics, CNES/Airbus DS, USDA, USGS, AeroGRID, IGN, and the GIS User Community.

The measurements of land movements available from permanent GPS stations and SAR-based interferometry also highlight a complex picture of the ground dynamics of the area (Fig. 6). The average magnitude and direction of the horizontal displacements provided by each GPS stations over the last decade are shown in Fig. 6A. SAR interferometry provides a much more detailed picture of the displacements, even if only in the west-east direction (Fig. 6B) because of the satellite movement along a polar (i.e., north-south) orbit. The west-east displacement map, which has been obtained by properly combining Cosmo-SkyMED images acquired over the 2011–2014 period in ascending and descending mode, reveals that the horizontal movements are characterized by a very complex pattern with uneven values. In particular, significant eastward movements (up to 4–5 mm/yr) are detected only between the Neto river delta and Capo Colonna, and in part of the Tacina river valley to the west (Fig. 6B). Patched positive (i.e., eastward) and negative (i.e. westward) displacements are measured elsewhere, with relatively small values (in general less than 1–2 mm/yr). Interpolation of the south-north component obtained by GPS data partly overcomes the SAR mapping restriction (Fig. 6A) and shows a regional trend with values smaller than 1 mm/yr. In the northern and central parts of the basin the rates are even smaller.

Therefore, the updip termination of the sedimentary body that underwent seaward migration remains obscure, with both GPS and SAR data highlighting that the Messinian to Pleistocene succession does not glide radially toward the Ionian Sea, as proposed in previous studies^17^.

## Discussion

The present study proves that the Crotone Swell is a tens of km-scale megalandslide up to 1.5 s TWT (ca. 1.6 km) thick subjected to gravity gliding processes^1^. This is revealed by the presence of a basal detachment surface dipping seaward of 3–4°, which connects an extensional updip domain, consisting of seaward-dipping normal faults, with a compressional downdip domain (Figs 2 and 3A,B). Such an interpretation contrasts with previous ones that described the Crotone Swell as the result of a SW-verging tectonic-driven thrust^27,34^.

**Figure 7:**
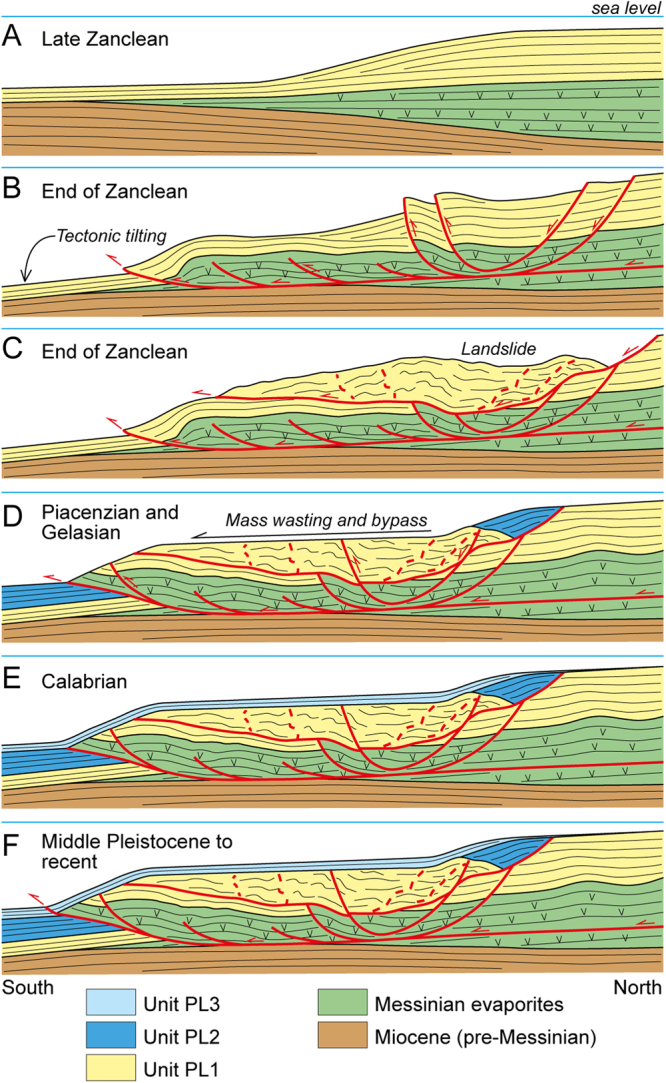
Inferred evolution of the seaward part of the Crotone megalandslide (not to scale), inspired by the F75_66 seismic profile (Fig. 2B). After a relatively tranquil phase of shelf margin progradation during Zanclean time (**A**), a tectonic event between Late Zanclean and Early Piacenzian (see text) led to tilting and triggered the gravity-driven movement (**B**). A minor landslide formed on top of the already moving megalandslide, leading to an exposed scar landward (**C**). Piacenziant to Gelasian deposits accumulate above the scar and the proximal part of the minor landslide, as well as in the deep basin, during a long-term phase of relatively slow movement of the megalandslide (**D**). A continuous sediment drape accumulated during an inferred phase of paucity of movement during Calabrian time (**E**). Since middle Pleistocene, the movement of the megalandslide has reactivated with modest rate, and still continues today (**F**).

A novelty of the present study is the discovery of a gravitational collapse nested in the main gravitational body and involving Messinian and Zanclean deposits (Units MES and PL1) (Figs 1C, 2B,C and 3A,B). This collapse is marked by a composite scar (surface SC) that is sealed by undisturbed Piacenzian-Gelasian deposits (Unit PL2) in its updip sector and tends to approach the seafloor landward (Figs 1C, 2B,C and 3A,B). This observation, together with the evidence that almost all structures are draped by Quaternary sediments (Unit PL3; Figs 2 and 3A,B), suggests that the seaward movement of the whole megalandslide started between Late Zanclean and Early Piacenzian times and continued until roughly Late Gelasian (Figs 2 and 7A–D), as demonstrated by local minor gravity-related faults affecting the surface SC and the deformed landmass in the seaward sector (Fig. 2B). Moreover, since the formation of the surface SC postdated the Unit PL1 and predated the Unit PL2 (Figs 2B, 3B and 7C,D), the gravitational collapse locally involving the upper part of the megalandslide, and in general the onset of the large-scale movement of the latter, were probably rapid. In contrast, the large hiatus associated with the surface bounding the base of the Unit PL3 in slope settings, suggests that the whole megalandslide continued to glide relatively slowly above the basal detachment surface until Late Gelasian (Figs 2 and 7D). This produced the observed km-scale seaward dislocation associated with the main gravity-driven thrust and prevented the accumulation of the Unit PL2 due to substrate instability, implying also that the accumulation of the Unit PL2 in basinal settings was contemporaneous to the progressive seaward gliding of the whole landmass (Fig. 7D). Once the movement of the megalandslide ceased, the Calabrian to late Pleistocene Unit PL3 started to accumulate and later was in turn affected by a reactivation of the gravitational phenomenon (Figs 2 and 7E,F). The hm-scale dislocation of the Unit PL3 by the main thrust at the base of the slope points to a very modest post-Gelasian activity, although the involvement of the seafloor suggests that it is still active (Figs 3A and 4).

The inferred Late Zanclean-Early Piacenzian age for the onset of the activity of the megalandslide (Fig. 7B,C) strongly suggests that a well-known mid-Pliocene (ca. 3.7 Ma) compressional-transpressional tectonic event, which affected the whole Calabrian Arc and produced thrusting and basin inversions^23,27,30,37^, was the triggering mechanism. In contrast, the end of the main seaward gliding of the megalandslide during Gelasian time (Fig. 7D,E) is roughly coincident with an episode of increased tectonic subsidence and basin collapse started at ca. 2.2 Ma, which involved the currently exposed part of the Crotone Basin and is reflected by the opening of the Marsili Basin in the Tyrrhenian Sea^27,30^. This tectonic episode may have led to deepening and to a decrease of the local slope toward the Ionian Sea, which halted the seaward gliding of the megalandslide. The Quaternary reactivation of the gravitational phenomenon, affecting also the Unit PL3 (Fig. 7F), was possibly triggered by the regional uplift involving the Crotone Basin since Middle Pleistocene (starting from ca. 0.45 Ma^27^), which reached maximum rates just south of the Crotone city^32^ and may have increased the dip of the seafloor toward the Ionian Sea. Alternatively, the reactivation might have been triggered by another known compressional-transpressional tectonic event occurred at ca. 1.1 Ma^27,30,31^. However, since the gravitational phenomenon seems to be still active, as demonstrated by the seafloor disturbance of the main thrust (Figs 3A and 4), a more recent reactivation of the megalandslide associated with the regional uplift is favored. Moreover, the concomitant relative sea-level drops associated with the Late Quaternary, high-magnitude glacio-eustatic changes may have favored the gravitational movement.

In contrast to what was postulated by Minelli, *et al*.^17^, who assume a radial gliding toward the Ionian Sea, present data indicate that the gravity-driven structures have SE-ward vergence (Fig. 2), which was controlled by the reactivation with dextral shear sense of the NW-trending Petilia-Sosti shear zone, thus representing the southwestern boundary of the megalandslide (Fig. 1B,C). At present, a similar role of transfer fault with respect to the megalandslide is not documented for the shear zone that represents the northern boundary of the Crotone Basin (i.e., the Rossano-San Nicola shear zone, Fig. 1B,C). The non-homogeneous eastward movements spanning from less than 0.5 to about 5 mm/yr provided by interferometric SAR-data, together with the negligible N-S velocity field obtained by GPS data (Fig. 6A,B), suggest that active structures and/or local gravitational phenomena involve the onshore sector, further denying a radial gliding of the structure.

The evidence discussed above allows us to estimate the rate of the seaward movement of the megalandslide. Assuming a minimum shortening associated with the thrust front of 10 km (based on the F75_68 seismic profile), and that the long-term gliding initiated at 3.7 Ma and terminated at 2.2 Ma, a minimum displacement rate of 6.7 mm/yr to the SE is expected during this phase. However, the displacement rate of the megalandslide may have been even locally greater, in particular if the observed additional shift of ca. 8 km to the SE of the thrust front just east of the Petilia-Sosti shear zone (i.e., east of the F75_68 seismic profile; Fig. 1B) is mainly related to differential gravity-driven gliding rather than regional strike-slip tectonics. In contrast, assuming that the Quaternary reactivation of the gravitational phenomenon started at ca. 0.45 Ma, at the onset of the regional uplift, and taking into account that the maximum offset of the Unit PL3 is ca. 500 m (Fig. 2), a significantly more modest displacement rate of ca. 1 mm/yr would be associated with the most recent gliding phase.

**Figure 8:**
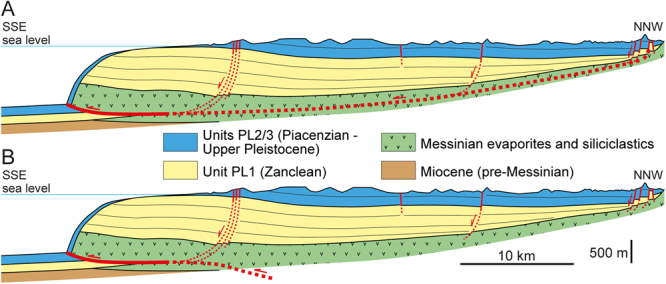
Simplified onshore-offshore geologic section (Fig. 1B for location) reporting two contrasting models to explain the development of the Crotone megalandslide. For a better reading, the recognized seismic units (Figs 2 and 3) are shown. (**A**) In the hypothesis that the gravity-driven movement involves the whole Crotone Basin, the basal detachment surface would connect the frontal thrust with seaward-dipping normal faults found to the north. Note that the dislocations associated with the normal faults are unable to justify the km-scale displacement associated with the frontal thrust. (**B**) Alternatively, the frontal thrust might have initiated as tectonic thrust during the mid-Pliocene tectonic event (see text) and be rooted at depth via a NW-ward dipping ramp in more landward position. In this case, only the offshore part of the sedimentary succession, corresponding to the Crotone Swell, would have been reactivated as gravitational collapse, and no detachment surface would be present in most of the onshore part of the basin fill.

A peculiar aspect is represented by the landward prosecution of the main detachment surface at the base of the megalandslide, and therefore the scale of the seaward-moving landmass. Assuming that the detachment surface led to the seaward gliding of the whole onshore part of the Messinian to Plio-Pleistocene deposits of the Crotone Basin (Fig. 8A), as advocated previously^17^, it is remarkable that this sedimentary succession remained nearly undeformed (Fig. 3C), excepting for some m- to hm-scale high-angle normal faults found in the northern part of the basin and in the Crotone peninsula (Fig. 1B). This situation would be very unusual, as the body of giant slides is commonly rather deformed^15,16^. The onshore part of the detachment surface would lie near the base of the Messinian strata (Fig. 8A), probably at the halite layer, and would be parallel to the bedding and impossible to discriminate from stratal surfaces in seismic profiles (Fig. 3B,C). The evidence of salt diapirism in the northern part of the basin might be associated with extension of the updip domain and to a local mobile layer. However, the non-homogeneous GPS and SAR data (Fig. 6) suggest that at least at present, an overall SE-ward migration of the onshore part of the basin fill does not occur. Moreover, the lacking evidence of chaotic structures linked to a mega-detachment zone in the hypothetical onshore updip domain (Fig. 8A), which could justify the horizontal displacement observed offshore, makes the situation even more unusual and allows some speculations.

A first possibility is that the Messinian to Zanclean part of the succession shifted to the SE of several km, aided by the halite layer and driven by the two NW-trending shear zones, just since the mid-Pliocene tectonic event, leaving behind an area deprived of such a succession. In fact, in the Cirò area, north of Crotone, the Piacenzian succession directly lies on Tortonian deposits without interposed Messinian and Zanclean deposits^38^, but such a situation is not recognizable along the NW margin of the basin. Following a second alternative hypothesis, most of the shortening associated with the main offshore thrust might be related to compressional tectonics during the mid-Pliocene tectonic event, and then such a structure might have been reactivated later as gravitational collapse only in the offshore sector (Fig. 8B). In this latter hypothesis, the mid-Pliocene thrust would be rooted at depth via a NW-ward dipping ramp in more landward position, and no detachment surface in the Messinian succession would be present in most of the onshore part of the basin fill (Fig. 8B). Only the seaward-dipping faults found in the Crotone peninsula would be linked to the detachment surface (Fig. 8B). The availability of more subsurface data would confirm one of the two hypotheses presented above as well as provide a new interpretation.

Finally, although still active, the Quaternary seaward migration of the subaqueous part of the megalandslide, in the order of only few hundreds of meters in the last 0.5 Ma, seems to be very modest if compared to its Pliocene counterpart, and therefore it is expected not to significantly affect human activities in the onshore sector of the basin. However, recent gravity-driven phenomena along the modern Calabrian slope may represent the testimony of the modern, low-rate seaward migration of the large-scale landmass, an aspect to consider for any offshore activity.

## Methods

2D multichannel seismic profiles and well logs used in this study (Figs 2, 3 and 5) have been made available by the Ministry of the Economic Development in the framework of the project “Visibility of Petroleum Exploration Data in Italy” (ViDEPI) (http://unmig.sviluppoeconomico.gov.it/videpi/).

Concerning the F75_66, F75_68 and F75_115 seismic profiles (Fig. 2 and Supplementary Material Fig. S1), the seismic images were converted into SEG-Y files by using a home-made code that includes parts of Seismic Unix software^39^. A Kirchhoff post-stack time migration was then applied to the data by using information about the velocities obtained for application of the normal move out labelled in the original figure. In order to obtain a better seismic image and avoiding artifacts such as smiles, the velocity fields were decreased of 20%. A band pass filtering was then applied to remove the random noise produced by migration.

Bathymetric data (Fig. 1B,C) and the OGS subbottom profile MAGIC31309_020 (Fig. 4) were acquired by OGS in the frame of the MaGIC (Marine Geohazards along the Italian Coasts) project. The subbottom profile was acquired during a geophysical campaign onboard of the R/V OGS Explora in April 2009 using CHIRP II Benthos CAP-6600 system, comprising 16 hullmounted transducers operating at frequencies of 2–7 kHz (submetric vertical resolution).

All spatial data were gathered in a digital GIS. Seismic facies and horizons were identified on seismic reflection profiles and correlated to stratigraphic data available from wells. The conversion from time to depth in seismic profiles was done by comparing seismic data with closer wells and by using average time-depth relationships based on available interval velocities. On these basis, main seismic units, stratigraphic surfaces and faults were defined and their significance was interpreted.

Land movements are based on the Persistent Scatterer Interferometry (PSI) products made available by the Italian Ministry of the Environment and Protection of Land and Sea (http://www.pcn.minambiente.it). Specifically, we used COSMO-SkyMed frames 60, 62, 63, 64, consisting of a stack of 45 and 40 stripmap images acquired between 2011 and 2014 in ascending and descending mode, respectively. For the specific PSI processing, refer to Costantini, *et al*.^40^.

The interferometric products (Fig. 6B) have been calibrated and de-flattened by the use of correction planes^41,42^, modeled through the velocities time series data recorded by PIPA, MEL2, CCRI, CROT, CUTR, STSV and SERS permanent GPS stations available from MAGNET GPS network (http://geodesy.unr.edu). Firstly, SERS (Fig. 6B), which is located outside the most part of the Crotone megalandslide, was selected to setup a local reference frame^43^. Secondly, the GPS velocities were projected along the Line-of-Sight (LOS) direction for each interferometric product. Finally, the average vertical (*u*_*z*_) and east-west (*u*_*x*_) ground movements (Fig. 6B) referred to SERS were computed by solving the linear system of equations (1):

1 $$\{\begin{array}{rcl}{u}_{asc} & = & {u}_{x}{n}_{x,asc}+{u}_{z}{n}_{z,asc}\\ {u}_{desc} & = & {u}_{x}{n}_{x,desc}+{u}_{z}{n}_{z,desc}\end{array}$$

where *u*_*asc*_ and *u*_*desc*_ are the components of the displacement vector **u** along the LOS ascending and descending direction; *n*_*x,asc*_, *n*_*z,asc*_, *n*_*x,desc*_, *n*_*z,desc*_ are the direction cosines identifying the satellite LOS vector for ascending and descending acquisition, respectively. Because of the different localization of reflecting Point Targets (PTs) in the two modes, the **u** has been resampled on a regular cell-grid of 50 × 50 m^2^ averaging the movement of the PTs belonging to the same cell^44,45^.

## Electronic supplementary material

Supplementary material
