# Supplementary material for: The Crotone Megalandslide, southern Italy: Architecture, timing and tectonic control
Source: Sci Rep. 2018 May 17;8:7778. doi: 10.1038/s41598-018-26266-y (PMC5958054; doi:10.1038/s41598-018-26266-y)
Supplement: Supplementary file 1 — Supplementary material [file 41598_2018_26266_MOESM1_ESM.pdf]

Supplementary Material for:

The Crotone Megalandslide, southern Italy: Architecture, timing and tectonic control

Massimo Zecchin[1], Flavio Accaino[1], Silvia Ceramicola[1], Dario Civile[1], Salvatore Critelli[2], Cristina Da Lio[3], Giacomo Mangano[1], Giacomo Prosser[4], Pietro Teatini[3,5], Luigi Tosi[3]

[1](OGS) Istituto Nazionale di Oceanografia e di Geofisica Sperimentale, Borgo Grotta Gigante, 42/c, 34010 Sgonico, Trieste, Italy. [2]Dipartimento di Biologia, Ecologia e Scienze della Terra, Università della Calabria, 87036 Arcavacata di Rende (CS), Italy. [3]Institute of Marine Sciences, National Research Council, Arsenale - Tesa 104, Castello 2737/F, 30122, Venezia, Italy. [4]Dipartimento di Scienze Geologiche, Università della Basilicata, Potenza, Italy. [5]Department of Civil, Environmental and Architectural Engineering, University of Padua, via Marzolo 9, 35121 Padova (PD), Italy. Correspondence and requests for materials should be addressed to M.Z. (email: mzecchin@inogs.it) or L.T. (email: luigi.tosi@ismar.cnr.it)

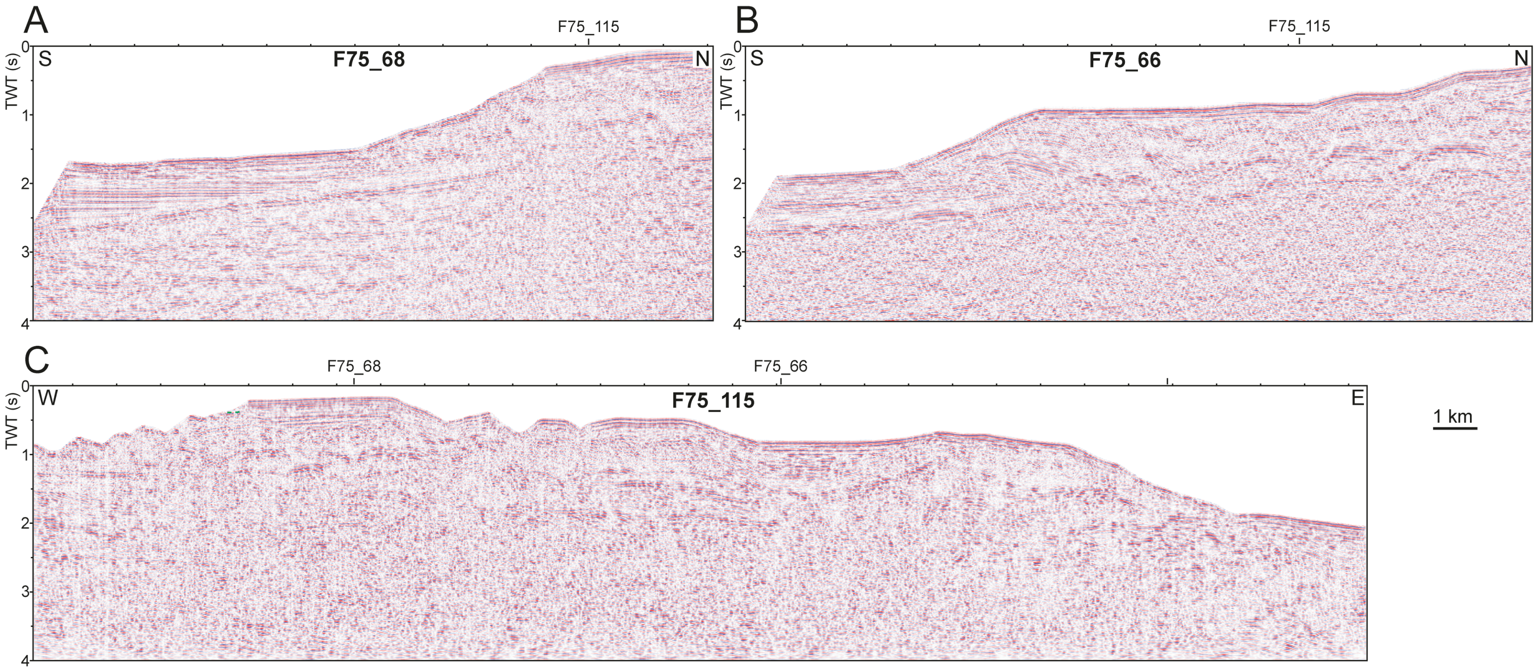

Figure S1. Uninterpreted seismic profiles F75_66, F75_68 and F75_115 (see Fig. 1 for location and Fig. 2 for the interpreted version).
